# Advances With Non-coding RNAs in Neuropathic Pain

**DOI:** 10.3389/fnins.2021.760936

**Published:** 2021-12-23

**Authors:** Cheng Hu, Menglin He, Qian Xu, Weiqian Tian

**Affiliations:** Affiliated Hospital of Nanjing University of Chinese Medicine, Jiangsu Province Hospital of Chinese Medicine, Nanjing, China

**Keywords:** neuropathic pain, non-coding RNAs, miRNA, lncRNA, circRNA

## Abstract

Neuropathic pain (NP) is one of the most common types of clinical pain. The common causes of this syndrome include injury to the central or peripheral nervous systems and pathological changes. NP is characterized by spontaneous pain, hyperalgesia, abnormal pain, and paresthesia. Because of its diverse etiology, the pathogenesis of NP has not been fully elucidated and has become one of the most challenging problems in clinical medicine. This kind of pain is extremely resistant to conventional treatment and is accompanied by serious complications. Non-coding RNAs (ncRNAs), such as microRNAs (miRNAs), long non-coding RNAs (lncRNAs), and circular RNAs (circRNAs), contribute to diverse biological processes by regulating the expression of various mRNAs involved in pain-related pathways, at the posttranscriptional level. Abnormal regulation of ncRNAs is closely related to the occurrence and development of NP. In this review, we summarize the current state of understanding of the roles of different ncRNAs in the development of NP. Understanding these mechanisms can help develop novel therapeutic strategies to prevent or treat chronic pain.

## Introduction

The International Association for the Study of Pain Taxonomy defines neuropathic pain (NP) as “pain caused by a lesion or disease of the somatosensory nervous system” (Widerstrom-Noga, 2017). The pathogenesis of NP includes peripheral and central mechanisms. The peripheral mechanism includes the abnormal discharge of injured peripheral afferent fibers, the electrical and sympathetic sensory coupling induced by the mixed transmission of neurons, and the increased excitability of adjacent undamaged fibers. The central mechanism includes the sensitization of spinal dorsal horn (SDH) neurons, decline of central inhibitory intermediate neurons, the sprouting of A_β_ fibers, activation of the descending facilitation system, and sensitization and activation of glial cells in the higher brain center. Many kinds of peripheral or central nervous system disorders can cause chronic pain. Diabetes, hypothyroidism, uremia, nutritional deficiency, and chemotherapy drugs (vincristine, paclitaxel, cisplatin, etc.) can lead to neuralgia, Guillain–Barre syndrome, post-herpetic neuralgia, progressive neuromuscular atrophy, complex regional pain syndrome type I, and ischemic neuropathy. The comorbidities in patients with NP include poor sleep, depression, and anxiety. Thus, NP greatly impairs quality of life and has a high economic impact on society. Despite the widespread interest, the pathogenesis of NP is not well understood to date, which in turn has resulted in the lack of effective clinical treatment options. It has become a difficult and hot topic in pain research (von Hehn et al., 2012; Yan et al., 2018a).

NP has become a universal healthcare issue affecting a wide range of people across the world. In the United States, NP costs an estimated $600 billion a year in healthcare and productivity losses (Gilron and Dickenson, 2014; Holmes, 2016). At present, the commonly used clinical treatment methods include local nerve block, sympathetic block, physiotherapy, opioid anesthetics, anti-infectives, antidepressants, and/or anxiolytics. In spite of these various options, pain management remains a challenge. To develop newer and better treatment strategies, it is necessary to gain a better understanding of the molecular mechanisms that lead to NP (Yan et al., 2017).

RNA has always been considered as a simple and intermediate component of gene expression, because it is transcribed from DNA and then translated into proteins in cells. However, the mammalian genome encodes not only protein-coding RNA but also a large number of non-coding RNAs (ncRNAs) (Lutz et al., 2014). ncRNAs play an important role in physiological and pathological NP processing. Specific processes within the cellular components of the NP triad that includes neurons, immune cells, and glia as well as the communication pathways among them are controlled by specific microRNAs (miRNAs) (Plassais et al., 2016; Liu et al., 2017; Shao et al., 2018; Vieira et al., 2018; Kalpachidou et al., 2020). A few studies have shown that miRNAs modulate inflammatory and NP conditions and therefore can be used as biomarkers of these conditions (Andersen et al., 2014; Gaudet et al., 2018). Long non-coding RNAs (lncRNAs) and circular RNAs (circRNAs) regulate mRNA expression in the progression of NP *via* their cross talk with miRNAs (Song et al., 2020).

In this article, we review the existing evidence for the changes in the three types of ncRNAs—miRNA, lncRNA, and circRNA—in pain-related regions after nerve injury. We also discuss how peripheral noxious stimuli induce such changes and explore potential mechanisms of how expressional changes in dorsal root ganglion (DRG), injured nerve, or spinal cord dorsal horn ncRNAs contribute to the development and persistence of NP. The known ncRNAs play a key role in gene regulation. Harmful peripheral stimuli can cause changes in the expression of ncRNAs which are related to hyperalgesia in NP (Bali and Kuner, 2014).

## Micrornas in Neuropathic Pain

MiRNAs are small (21–23 nucleotides) ncRNA molecules that bind to the 3′ untranslated region (UTR) of the target mRNA at a specific sequence and act as posttranscriptional regulators of gene expression. The regulatory mechanisms include silencing mRNA expression or degrading the transcript in various biological processes or states (Aldrich et al., 2009). Mounting evidence shows that miRNA is strongly associated with pain pathways from primary afferent pain receptors, DRG, spinal cord, and brain regions in different NP models (Sun et al., 2012; Sakai et al., 2013; Park et al., 2014; Pan et al., 2016; Peng et al., 2017). Some cases of neuropathic, inflammatory, and cancer pain can be prevented or reversed *via* the regulation of miRNA expression (Bali et al., 2013; Pan et al., 2014; Jiang et al., 2016). The possible role of miRNAs in gene regulation relative to NP is discussed below.

In partial sciatic nerve ligation (pSNL) induced NP, the expression of chemokine CXC receptor 4 (CXCR4) was higher and miRNA-23a-3p (miR-23a) expression was significantly lower in spinal glial cells. MiR-23a can bind directly to the 3′ UTR of CXCR4 mRNA. Knockdown of miR-23a or overexpression of CXCR4 in mice can increase the thioredoxin-interacting protein (TXNIP), which is related to the induction of NOD-like receptor protein 3 (NLRP3) inflammasome in pSNL mice. Inhibition of TXNIP can reverse pain behaviors induced by pSNL. In short, miR-23a regulates NP in pSNL mice by directly targeting CXCR4, *via* the TXNIP/NLRP3 inflammasome axis (Pan et al., 2018). In mice with chronic constriction injury (CCI), miR-381 can inhibit the development of NP by targeting the high-mobility group box 1 protein (HMGB1) and CXCR4 (Zhan et al., 2018). Peripheral nerve injury activates the expression and release of C–X–C motif chemokine 13 (CXCL13) from spinal cord neurons to have an effect on the CXCR5 in astrocytes. The induced astrocytes activate to maintain NP. MiR-186-5p negatively regulates CXCL13. The downregulation of miR-186-5p in spinal cord neurons in SNL leads to the upregulation of CXCL13, which leads to NP (Jiang et al., 2016).

Cav1.2 consists of the L-type calcium channel (Cav1.2-LTC) and plays a key role in chronic NP. MiR-103 can simultaneously affect the expression of the three subunits forming Cav1.2-LTC in a novel integrative regulation. MiR-103 plays a bidirectional and integrated regulatory role in Cav1.2-LTC, which is likely to be the primary cause of chronic pain sensitization. In fact, downregulation of miR-103 levels can induce hyperalgesia in animals (Favereaux et al., 2011). MiR-17-92, a miRNA cluster with six different elements, cannot only downregulate the expression of potassium channels but also reduce extracellular potassium currents, especially A-type currents. A combination of potassium channel regulators can relieve the mechanical allodynia caused by nerve injury or overexpression of miR-17-92. The miR-17-92 cluster seems to co-regulate the function of multiple voltage-gated potassium channel subunits (Sakai et al., 2017). MiR-7a is a pivotal functional miRNA, which maintains the stage of NP in rats by regulating the excitability of neurons. In the late stage of NP, miR-7a is a noticeable change miRNA in the injury DRG. The downregulation of miR-7a in itself is not sufficient to lead to pain-related behaviors in rats. MiR-7a targets the β2 subunit of the voltage-gated sodium channel. A decrease in miR-7a leads to the increase in β2 subunit protein expression. The increased expression of the β2 subunit results in long-term hyperactivity of the injured neurons and persistence of pain behaviors (Sakai et al., 2013). In SNL-induced NP, miR-30b can directly target SCN3A 3′UTR, alleviating NP mainly *via* inhibiting the expression of voltage-gated sodium channel Nav1.3 in DRG neurons and spinal cord (Su et al., 2017).

SNL results in the downregulation of miRNAs such as miR-183 family in ipsilateral lumbar 5 (L5) DRG. In addition, the nerve injury leading to mechanical hypersensitivity is accompanied by the general redistribution of miRNA around DRG neurons with and without myelin sheath (Aldrich et al., 2009). The expression levels of miR-124a and miR-155 were higher and the histone deacetylase sirtuin1 (SIRT1) levels were lower in NP patients compared to those in healthy volunteers. In fact, the two miRNAs were negatively correlated with SIRT1 transcription level and were found to be direct inhibitors of SIRT1 in human CD4 + cells. SIRT1, targeted by these two miRNAs, can increase the expression level of Foxp3, which leads to an increase in anti-inflammatory regulatory T cells (Tregs) (Heyn et al., 2016). It was found that in DRG, IL-6 increased the expression of miR-21, miR-431, and miR-511-3p. The expression of miR-21 in exosomes extracted from blood was higher after spared nerve injury (SNI), which may play a partial role in NP (Hori et al., 2016). There is evidence which shows that miRNA-146a-5p in DRG and SDH can regulate CCI-induced NP by inhibiting interleukin-1 receptor-associated kinase 1 (IRAK1) and tumor necrosis factor receptor-associated factor 6 (TRAF6) in toll/interleukin-1 receptor (TIR) signaling pathway (Wang Z. et al., 2018). MiR-146a-5p can alleviate NP by inhibiting TRAF6 and its downstream JNK/CCL2 signal pathway (Lu et al., 2015). MiR-218 negatively mediates the signal transducer and activator of transcription 3 (STAT3) signaling pathway *via* regulating the suppressor of cytokine signaling 3 (SOCS3) expression, and miR-221 inhibits nuclear factor-kappa B (NF-kB) and p38 mitogen-activated protein kinase (p38MAPK) signaling pathway by regulating the SOCS1 level to alleviate CCI-induced NP and neuroinflammation (Li and Zhao, 2016; Xia et al., 2016). MiR-223 can inhibit the activity of NLRP3 inflammasomes by downregulating NLRP3, which in turn reduces morphine tolerance in rats with CCI-induced NP. MiR-34c can inhibit the development of NP primarily by inhibiting NLRP3-regulated neuroinflammation (Xie X.J. et al., 2017; Xu et al., 2019). MiR-26a-5p regulates the development of NP by targeting MAPK6 in CCI-treated rats (Zhang et al., 2018a). In rats with NP, CCI resulted in a time-dependent downregulation of miRNA-1 in sciatic nerve (SN), while miRNA-1-targeted protein brain-derived neurotrophic factor (BDNF), connexin 43 (Cx43) in SN, and DRG were upregulated (Neumann et al., 2015).

In rats with SN injury, the expression of miR-30c-5p positively correlated with NP. MiR-30c-5p is related to the bone morphogenetic protein and membrane-bound inhibitor (BAMBI), a transforming growth factor-β (TGF-β) pseudo receptor (Tramullas et al., 2018). The abnormal expression of miR-132-3p is strongly associated with human NP. In the SNI model, miR-132-3p levels were increased leading to the upregulation of AMPA receptor subunits GluA1 and GluA2 in the spinal cord (Leinders et al., 2016).

Upregulation of miR-200b and miR-429 expression in CCI mice can inhibit neuroinflammation by inhibiting inflammatory cytokines. MiR-200b/miR-429 can be an important regulator of NP by targeting zinc finger E box binding protein-1 (ZEB1) (Yan et al., 2018c). In the CCI rats, miR-378 was downregulated, and the overexpression of miR-378 provided significant pain relief. Enhancer of zeste homolog 2 (EZH2) is the downstream gene of miR-378 and is negatively regulated by miR-378 (Gao et al., 2021). The methyl CpG-binding protein 2 (MeCP2) plays a key role in neuron differentiation, neural development, and synaptic plasticity by modulating CREB-miR-132 to relieve acute and chronic pain (Zhang et al., 2015). Inhibition of the mTOR or VEGF receptor can significantly reduce NP, and the upregulation of miR-183 can suppress the AMPA receptor by inhibiting the mTOR/VEGF pathway (Xie X. et al., 2017). In CCI mice, overexpression of miR-233 inhibits the polarization and recruitment of macrophages *via* the NLRP3/IL-1β pathway to ameliorate NP (Zhu et al., 2021). In spinal cords of rats with CCI-induced NP, miR-122-5p expression was decreased. Overexpression of miR-122-5p can prevent NP behavior. Pyruvate dehydrogenase kinase 4 (PDK4) is a downstream protein, and inhibition of its expression by miR-122-5p attenuates NP (Wan et al., 2021). MiR-142-3p expression was higher, and AC9 expression was lower in rats with CCI-induced NP. MiR-142-3p inhibition could increase the expression of AC9 and cAMP and further increase the activity of AMPK (Li et al., 2021).

In the DRG of oxaliplatin-induced NP rats, miR-30b-5p expression decreased meanwhile the mRNA and protein expression levels of Na_v_1.6 increased. MiR-30b-5p contributes to NP through Na_v_1.6 downregulation (Li L. et al., 2019). MiR-15b was increased in the oxaliplatin-induced NP, β-site amyloid precursor protein-cleaving enzyme 1 (BACE1) was a target of miR-15b, and miR-15b and BACE1 were negatively correlated in NP (Ito et al., 2017). It was found that NP caused by paclitaxel or SNL significantly damaged the GABAergic synaptic function of SDH neurons by reducing the expression of GAD67. The expression levels of miR-500 increased in NP and regulated GAD67 by targeting specific sites of the GAD1 gene in the dorsal horn. Downregulation of miR-500 could rescue the GABAergic synapses in the SDH neurons and attenuate the sensitized pain behavior in rats with NP (Huang et al., 2016). Intrathecal injection of miRNA-138 can alleviate NP in rats with a pSNL, which may be achieved by suppressing the toll-like receptor 4 (TLR4) and macrophage inflammatory protein-1 alpha (MIP-1α)/C-C chemokine receptor type 1 (CCR1) signaling pathways (Jin et al., 2021). The downregulation of NADPH oxidase 4 (NOX4) caused by miR-23b can effectively induce TXNL1, GPX3, and SEPN1 gene expression after spinal cord pain induction. MiR-23b plays an important role in the improvement of NP caused by spinal cord injury (SCI) by inactivating NOX4. In animals with NP, the exogenous expression of miR-23b and NOX4 antibody effectively reduced pain symptoms and increased the survival of GABAergic neurons and the recovery of GAD expression in SCI. The expression of exogenous miR-23b in neuropathic lesions can effectively control inflammatory symptoms, including microglial infiltration and inflammatory factor secretion, and reduce ROS-mediated GABAergic nerve cell death (Im et al., 2012). MiR-183 modulates the expression of MAP3K4, which in turn leads to the downregulation of inflammatory factors, and cyclooxygenase-2 (COX-2) which slows NP progression. In summary, the study stated that miR-183 was a part of the negative regulatory effector group, which could alleviate NP by targeting MAP3K4 (Huang and Wang, 2020). MiR-21 can interact with toll-like receptor 8 (TLR8) in lysosome as an endogenous ligand, inducing extracellularly regulated protein kinase (ERK) activation and production of inflammatory mediators. It further enhances the excitability of neurons and contributes to the persistence of NP (Zhang et al., 2018c).

In diabetic neuropathic pain (DNP), the miR-190a-5p level was decreased and SLC17A6 [the solute carrier family 17 (sodium-dependent inorganic phosphate cotransporter), member 6] was increased in the spinal tissue compared to that in the control group. Upregulation of miR-190a-5p and inhibition of SLC17A6 could significantly weaken the pain-related behavior and reduce IL-1β and IL-6 levels in DNP (Yang et al., 2017). Inhibition of miR-221 can reduce pain and decrease the expression of pain-related factors (bradykinin, prostaglandin E2, IL-6, IL-1β, and tumor necrosis factor alpha) through targeting SOCS3 in the DNP rat model (Wu et al., 2021). MiR-590-3p inhibits T cell infiltration by targeting Ras-associated protein 1A (RAP1A), thus ameliorating diabetic peripheral NP in animal models (Wu et al., 2020).

In some commonly used NP models, specific miRNAs are upregulated or downregulated along the pain pathway. The above part summarizes the imbalance of miRNA in pain model tissues and the role of related target genes in the mechanism of NP. MiRNAs associated with NP are listed in Table 1.

**TABLE 1:** miRNAs associated with neuropathic pain.

Models	miRNAs	Expression	Tissue	Target gene	References
pSNL	miR-23a-3p	↓	Spinal glial cells	CXCR4 TXNIP/NLRP3	Pan et al., 2018
CCI	miR-381	↑	Spinal cord tissues	HMGB1/CXCR4	Zhan et al., 2018
SNL	miR-186-5P	↓	Spinal astrocyte	CXCL13	Jiang et al., 2016
SNL	miR-103	↓	Spinal cord	Cav1.2-LTC	Favereaux et al., 2011
SNL	miR-17-92	↑	DRG	Voltage-gated potassium channel	Sakai et al., 2017
SNL	miR-7a	↓	DRG	Voltage-gated sodium channel	Sakai et al., 2013
SNL	miR-30b	↓	DRG/spinal cord	Voltage-gated sodium channel Nav1.3	Su et al., 2017
NP patients	miR-124a, miR-155	↑	Blood samples	SIRT1	Heyn et al., 2016
SNL	miR-183	↓	L5 DRG	T-Cell intracellular antigen 1 (TIA-1)	Aldrich et al., 2009
SNL	miR-21, miR-431, miR-511-3p	↑	DRG	IL-6	Hori et al., 2016
CCI	miR-146a-5p	↑	DRG/SDH	IRAK1/TRAF6	Wang Z. et al., 2018
SNL	miR-146a-5p	↑	Spinal cord	TRAF6/JNK	Lu et al., 2015
SCI	miR-218	↓	Spinal cord	SOCS3/p-P38	Li and Zhao, 2016
CCI	miR-221	↑	Spinal cord	SOCS1	Xia et al., 2016
CCI + tolerance morphine	miR-223	↓	Spinal cord	NLRP3	Xie X.J. et al., 2017
CCI	miR-34c	↓	Spinal cord	NLRP3	Xu et al., 2019
CCI	miR-26a-5p	↓	Spinal cord	MAPK6	Zhang et al., 2018a
CCI	miR-1	↓	SN/DRG	Cx43/BDNF	Neumann et al., 2015
SNI	miR-30c-5p	↑	Spinal cord/DRG/plasma/fluid cerebrospinal	BAMBI	Tramullas et al., 2018
DNP	miR-190a-5p	↓	Spinal dorsal horn	SLC17A6	Yang et al., 2017
DNP	miR-193a	↓	Spinal dorsal horn	HMGB1	Wu B. et al., 2019
DNP	miR-221	↑	Serum	SOCS3	Wu et al., 2021
DPNP	miR-590-3p	↓	DRG/T cells	RAP1A	Wu et al., 2020
SCI	miR-130a-3p	↑	Spinal cord	IGF-1/IGF-1R	Yao et al., 2021
CCI	miR-378	↓	Spinal cord	EZH2	Gao et al., 2021
pSNL	miR-138	↑	Spinal cord	TLR4 MIP-1a/CCR1	Jin et al., 2021
CCI	miR-223	↓	Spinal cord	NLRP3/IL-1β	Zhu et al., 2021
CCI	miR-122-5p	↓	Spinal cord	PDK4	Wan et al., 2021
CCI	miR-142-3p	↑	Sciatic nerve	cAMP/AMPK	Li et al., 2021
NP patients/SNI	miR-132-3p	↑	White blood cells/SN/DRG/Spinal cord	GluA1/GluA2	Leinders et al., 2016
CCI	miR-200b/miR-429	↑	Microglia from the Spinal cord	ZEB1	Yan et al., 2018c
SNI	CREB-miR-132	↓	Spinal cord	MeCP2	Zhang et al., 2015
CCI	miR-183	↓	Spinal cord horn	mTOR/VEGF	Xie X. et al., 2017
Oxaliplatin-induced NP	miR-30b-5p	↓	DRG	Na_v_1.6	Li L. et al., 2019
Oxaliplatin-induced NP	miR-15b	↑	DRG	BACE1	Ito et al., 2017
L5 ventral root transection/paclitaxel-induced NP	miR-500	↑	Spinal cord horn	GAD67	Huang et al., 2016
SCI	miR-23b	↓	Spinal cord	NOX4	Im et al., 2012
CCI	miR-183	↓	Spinal cord horn	MAP3K4/IL-6/IL-1β/COX2	Huang et al., 2020
SNI	miR-21	↑	DRG	TLR8	Zhang et al., 2018c

*NP, Neuropathic pain; DRG, dorsal root ganglion; pSNL, partial sciatic nerve ligation; SNL, sciatic nerve ligation; SNI, spared nerve injury; CCI, chronic constriction injury; SCI, spinal cord injury; DNP, diabetic neuropathic pain; CXCR4, chemokine CXC receptor 4; TXNIP, thioredoxin-interacting protein; NLRP3, NOD-like receptor protein 3; HMGB1, high-mobility group box 1 protein; CXCL13, C–X–C motif chemokine 13; Cav1.2-LTC, L-type calcium channel; SIRT1, sirtuin1; SDH, spinal dorsal horn; IRAK1, inhibiting interleukin-1 receptor-associated kinase 1; TRAF6, tumor necrosis factor receptor-associated factor 6; SOCS3, suppressor of cytokine signaling 3; SN, sciatic nerve; BDNF, brain-derived neurotrophic factor; Cx43, connexin 43; BAMBI, bone morphogenetic protein and membrane-bound inhibitor; ZEB1, zinc finger E box binding protein-1; MeCP2, methyl CpG-binding protein 2; NOX4, NADPH oxidase 4; TLR8, toll-like receptor 8; IL-1β, interleukin-1β; PDK4, pyruvate dehydrogenase kinase 4; RAP1A, Ras-associated protein 1A; SLC17A6, [solute carrier family 17 (sodium-dependent inorganic phosphate cotransporter), member 6]; MIP-1α, macrophage inflammatory protein-1 alpha and CCR1, C–C chemokine receptor type 1; AMPK, AMP-activated protein kinase; EZH2, enhancer of zeste homolog 2; BACE1, β-site amyloid precursor protein-cleaving enzyme 1.*

## Long Non-Coding RNA in Neuropathic Pain

LncRNA is a new kind of functional RNA discovered in the past decade. It is over 200 nucleotides long and is generally believed to play a crucial role in the regulation of gene expression. Its biological and molecular mechanisms are diverse and complex, which need further study (Zhang et al., 2018b). Rapidly accumulating evidence points out that close to 40% of lncRNA exists exclusively in the nervous system (Wu W. et al., 2019). LncRNAs have recently been found to be key modulators of neuronal functions, especially in NP (Li Z. et al., 2019).

In the SDH of mice with DNP, 1,481 differentially expressed (DE) lncRNAs and 1,096 DE mRNAs have been identified. It has been found that 289 neighboring and 57 overlapping lncRNA–mRNA pairs, including ENSMUST00000150952-Mbp and AK081017-Usp15, may be involved in DNP pathogenesis (Du et al., 2019). Liu C. et al. (2018) found that ERK and p38 MAPK signaling pathways may be involved in the processes by which lncRNA BC168687 siRNA alleviates DNP mediated by transient receptor potential vanilloid type 1 (TRPV1). Another study found that inhibition of lncRNA BC168687 expression may downregulate P2X purinoceptor (P2 × 7) receptor expression in satellite glial cells (SGCs) induced by high glucose and high free fatty acid environment (Liu C.L. et al., 2018). In type 2 DNP rats, siRNA of lncRNA NONRATT021972 can reduce the expression of P2 × 7 mRNA and protein in DRG and inhibit the activation of SGCs. In addition, NONRATT021972 siRNA treatment reduced the release of tumor necrosis factor-α (TNF-α), thereby inhibiting the excitability of DRG neurons and reducing NP (Liu et al., 2016). The expression of lncRNA uc 0.48 + was increased in the DRG of DNP rats, and serum of diabetic patients. The P2 × 3 protein and mRNA in DRG of diabetic rats were increased while uc 0.48 + siRNA treatment reverses the trend. Phosphorylation and activation of ERK1/2 also decreased. Therefore, uc 0.48 + siRNA treatment can alleviate DNP by inhibiting P2 × 3 receptor-mediated excitatory transfer in DRG (Wang et al., 2016). In the lumbar SDH of DNP mice, miR-193a was downregulated and HMGB1 expression was upregulated. Overexpression of miR-193a can inhibit HMGB1 expression and alleviate NP (Wu B. et al., 2019). LncRNA, NONRATT021972 increased in type 2 diabetes and was positively associated with NP score (Yu et al., 2017). In trigeminal ganglia, lncRNA uc 0.48 + can interact with the P2 × 7 receptor, upregulate P2 × 7 receptor expression, enhance ERK1/2 phosphorylation, and participate in pain transmission (Xiong et al., 2019). LncRNA, MRAK009713 could play a positive role in NP in rats through regulating the expression and function of the P2 × 3 receptor (Li et al., 2017).

In the SNL-induced NP mice, the DRGs of L5 were taken for microarray analysis of lncRNAs. From the fourth day of SNL treatment, H19 lncRNA increased significantly in L5 DRG and was detected mainly in non-neuronal cells. Consistent with this finding, the expression of H19 was upregulated in Schwann cells isolated from peripheral nerves, which might be related to NP (Iwasaki et al., 2019). In SNL model rats, lncRNA, PKIA-AS1 was significantly upregulated, while the inhibition of PKIA-AS1 slowed the progression of NP. In addition, overexpression of PKIA-AS1 can remarkably induce NP-like symptoms in uninjured rats. PKIA-AS1 can mediate SNL-induced NP through regulating the expression and function of cyclin-dependent kinase 6 (CDK6), which is essential for the occurrence and progression of neuroinflammation and NP. Therefore, PKIA-AS1 is expected to become a new therapeutic target for neuroinflammation associated with NP (Hu et al., 2019). The lncRNA Kcna2 antisense RNA, voltage-dependent potassium channel mRNA, can be regulated by transcriptional activation like mRNA. The upregulation of Kcna2 antisense RNA caused by nerve injury targets myeloid zinc finger protein 1, a transcription factor that binds to the Kcna2 antisense RNA gene promoter (Zhao et al., 2013).

In CCI rats, miR-136 could be the target of lncRNA colorectal neoplasia differentially expressed (CRNDE), and the loss of miR-136 caused NP by inducing neuroinflammation. In addition, miR-136 also targets IL-6R and regulates its expression. It has been confirmed that increased expression of IL-6R can be induced by CRNDE, and the CRNDE/miR-136/IL-6R axis had a significant effect on NP (Zhang D. et al., 2019). In CCI rats, lncRNA, DGCR5 relieves NP by sponging miR-330-3p and the downstream PDCD4 expression (Peng et al., 2019). In addition, lncRNA X inactive specific transcript (XIST) can positively regulate NP through regulating the expression of miR-137 and tumor necrosis factor alpha-induced protein 1 (TNFAIP1), which is involved in the regulation of inflammation *via* activating NF-kB activity (Zhao et al., 2018). Some research concluded that lncRNA, XIST can lead to NP development in rats through the inhibition of miR-544 and activation of STAT3 (Jin et al., 2018). Other research showed that XIST promotes the development of NP by inducing TLR5, which plays a role in the expression of miR-154-5p in CCI rats (Wei et al., 2018). It was found that miR-150 decreased significantly in CCI rats. It was postulated to be the target of XIST and had a negative correlation with XIST. Increased expression of miR-150 can significantly inhibit the expression of neuroinflammatory cytokines. In addition, ZEB1 is the direct target of miR-150. ZEB1 was found to be upregulated in CCI rats. Therefore, the XIST/miR-150/ZEB1 axis can be used as a therapeutic target to mitigate NP (Yan et al., 2018b). In the CCI rat model, the levels of some molecules, such as COX-2, TNF-α, interleukin-1β (IL-1β), and ZEB1 increased. Linc00657 can inhibit these molecules to alleviate NP. Inhibition of miR-136 has the same effect, indicating that ZEB1 is negatively correlated with miR-136 and linc00657 (Shen et al., 2019). LncRNA metastasis-associated lung adenocarcinoma transcript 1 (MALAT1) was significantly upregulated in CCI rats, and miR-206 was significantly downregulated. ZEB2 is the target of miR-206. The upregulation of miR-206 relieves NP caused by ZEB2 overexpression *in vivo* by inhibiting neuroinflammation. Inhibition of MALAT1 can slow the progression of NP through the miR-206/ZEB2 axis (Chen et al., 2019). In the CCI rat model, the expression of NEAT1 was significantly higher. MiR-381 decreased and HMGB1 increased indicating that NEAT1 can regulate the miR-381/HMGB1 axis to slow the progression of NP (Xia et al., 2018). Since natural antisense transcript (NAT) reduces the Nav1.7 current, it is highly likely that lack of NAT increases sodium current. This may lead to an increase in excitability of injury sensitive neurons, which in turn fine-tunes the response to pain stimuli (Koenig et al., 2015).

In SCI rats, inhibition of lncRNA plasmacytoma variant translocation 1 (PVT1) can alleviate NP by upregulating miR-186-5p and downregulating CXCL13/CXCR5 (Zhang P. et al., 2021). SCI rats showed higher nuclear paraspeckle assembly transcript 1 (NEAT1) expression compared to the sham group. Overexpression of NEAT1 enhanced the expression of inflammation factors. NEAT1 targeted and inhibited miR-128-3p, meanwhile miR-128-3p can target aquaporin-4 (AQP4) to regulate its expression (Xian et al., 2021). Compared to the control group, the miR-130a-3p expression was significantly upregulated in the spinal cord lesions of SCI rats. Downregulation of miR-130a-3p increased the expression of IGF-1 and IGF-1R (Yao et al., 2021).

Differential expressions of lncRNAs in NP models and lncRNA-targeted miRNAs or genes have been addressed in detail. LncRNAs play an important role in NP processes not only in neurons but also in non-neuronal cells related to the ion channel, neuroinflammation, purinoceptor, and so on. LncRNAs associated with NP are listed in Table 2.

**TABLE 2:** lncRNAs associated with neuropathic pain.

Models	lncRNA	Expression	Tissue	Target gene	References
SNL	Kcna2 antisense RNA	↑	DRG	Myeloid zinc finger protein 1	Zhao et al., 2013
DNP	lncRNA BC168687	↑	DRG	TRPV1	Liu C. et al., 2018
DNP	lncRNA BC168687	↑	SGCs of DRG	P2 × 7	Liu C.L. et al., 2018
DNP	lncRNA NONRATT021972	↑	DRG	P2 × 7	Liu et al., 2016
DNP	lncRNA uc 0.48 +	↑	DRG	P2 × 3/p-ERK1/2	Wang et al., 2016
DNP	lncRNA NONRATT021972	↑	Blood	TNF-a	Yu et al., 2017
SCI	lncRNA PVT1	↑	Spinal cord	miR-186-5p/CXCL13/CXCR5	Zhang P. et al., 2021
SCI	lncRNA NEAT1	↑	Spinal cord	miR-128-3p/AQP4	Xian et al., 2021
TN	lncRNA uc 0.48 +	↑	Trigeminal ganglia	P2 × 7/p-ERK1/2	Xiong et al., 2019
CCI	lncRNA MRAK009713	↑	DRG	P2 × 3	Li et al., 2017
SNL	H19 lncRNA	↑	L5 DRG	−	Iwasaki et al., 2019
SNL	lncRNA PKIA-AS1	↑	Spinal cord	CDK6	Hu et al., 2019
CCI	lncRNA CRNDE	↑	L4-L6 dorsal spinal cord	miR-136/IL-6R	Zhang D. et al., 2019
CCI	lncRNA DGCR5	↓	L4-L6 dorsal spinal cord	miR-330-3p/PDCD4	Peng et al., 2019
CCI	lncRNA XIST	↑	L4-L6 dorsal spinal cord	miR-137/TNFAIP1	Zhao et al., 2018
CCI	lncRNA XIST	↑	L4-L6 dorsal spinal cord	miR-544/STAT3	Jin et al., 2018
CCI	lncRNA XIST	↑	L4-L6 dorsal spinal cord	miR-154-5p/TLR5	Wei et al., 2018
CCI	lncRNA XIST	↑	L4-L6 dorsal spinal cord	miR-150/ZEB1	Yan et al., 2018b
CCI	lncRNA 00657	↑	L4-L6 dorsal spinal cord	miR-136/ZEB1	Shen et al., 2019
CCI	lncRNA MALAT1	↑	L4-L6 dorsal spinal cord	miR-206/ZEB2	Chen et al., 2019
CCI	linRNA NEAT1	↑	L4-L6 dorsal spinal cord	miR-381/HMGB1	Xia et al., 2018
CCI	SCN9A	↑	L4-L6 ipsilateral DRG		Koenig et al., 2015

*NP, Neuropathic pain; lncRNAs, long non-coding RNAs; DRG, dorsal root ganglion; SNL, sciatic nerve ligation; CCI, chronic constriction injury; DNP, diabetic neuropathic pain; TN, trigeminal neuralgia; SCI, spinal cord injury; CXCR5, chemokine CXC receptor 5; HMGB1, high-mobility group box 1 protein; CXCL13, C–X–C motif chemokine 13; STAT3, signal transducer and activator of transcription 3; ZEB1, zinc finger E box binding protein-1; TLR8, toll-like receptor 8; ERK, extracellular regulated protein kinases; TRPV1, transient receptor potential vanilloid type 1; P2 × 7, P2X purinoceptor; SGCs, satellite glial cells; L5, lumbar 5; CDK6, cyclin-dependent kinase 6; CRNDE, colorectal neoplasia differentially expressed; XIST, X inactive specific transcript; TNFAIP1, tumor necrosis factor alpha-induced protein 1; TNF-α, tumor necrosis factor-α; IL-6R, interleukin-6 receptor; MALAT1, metastasis-associated lung adenocarcinoma transcript 1; NEAT1, nuclear paraspeckle assembly transcript 1; AQP4, aquaporin-4.*

## Circular RNA in Neuropathic Pain

CircRNAs are produced by natural transcription of genomic DNA, but the bilateral exon transcription is covalently prevented. In most cases, they may be produced by non-covalent splicing. Most of the circRNAs are expressed in the cytoplasm and are very stable, indicating that they may have different functions from those of typical mRNA or lncRNA (Piwecka et al., 2017). CircRNAs are another type of ncRNAs that act as miRNA sponges and modulate gene expression through a circRNA–miRNA–mRNA pathway. In the widely used CCI NP models, differentially expressed circRNAs in sham-operated and NP rats were detected by using circRNA microarrays to elucidate the expression of circRNAs in NP (Hansen et al., 2013; Wilusz and Sharp, 2013; Cao et al., 2017).

Zheng et al. (2016) characterized the circRNA named circHIPK3, expressed abundantly from Exon2 of the HIPK3 gene. Silencing the circHIPK3 gene can dramatically inhibit the growth of human cells. Using the luciferase screening experiment, circHIPK3 was found to have 18 potential binding targets and bind nine miRNAs. Specifically, circHIPK3 binds directly to miR-124 and inhibits its activity (Zheng et al., 2016). Wang L. et al. (2018) found that circHIPK3 in the serum of DNP patients and the DRG of DNP rats was abundant. In type 2 diabetic patients, the upregulation of circHIPK3 was positively correlated with NP grade. Silencing circHIPK3 can alleviate NP in DNP rats, which is related to neuroinflammation. Further studies into its mechanism of action showed that circHIPK3 interacted with miR-124 and downregulated its expression. The miR-124 inhibitor can reverse the effect of circHIPK3 gene knockout on reducing NP and inhibiting neuroinflammation in DNP rats (Wang L. et al., 2018).

Spinal cord-specific circRNA, circAnks1a, increased in both the cytoplasm and the nucleus after SNL. In the cytoplasm, circAnks1a promotes the interaction between transcription factor YBX1 and transporter-1, thus facilitating the access of YBX1 to nucleus. The interaction between YBX1 and VEGFB promoter was promoted by specific RNA–DNA interaction. Cytoplasmic circAnks1a can also be used as miRNA sponge of miR-324-3p to enhance the translation of VEGFB mRNA. The upregulation of VEGFB stimulated dorsal horn neurons and promoted the pain behavior induced by nerve injury (Zhang S.B. et al., 2019).

In the CCI model, Cai et al. (2020) found that the expression level of ciRS-7 in the SDH was positively associated with the progress of NP; ciRS-7 is involved in the development of NP in part by upregulating autophagy and inflammation. In addition, ciRS-7 sponges to miR-135a-5p to regulate NP. When miR-135a-5p expression was inhibited, autophagy and inflammation were reduced, and pain was relieved (Cai et al., 2020). In CCI rat models, the expression levels of circ_0005075 were higher. Loss of circ_0005075 could repress neuroinflammation *via* targeting COX-2, IL-6, and TNF-α, while inducing IL-10 *in vivo*. Additionally, miR-151a-3p was significantly reduced in CCI rats and circ_0005075 reversed the repressive effect of miR-151a-3p on NP. In another study, NOTCH2 has been shown to induce a variety of intracellular responses correlated with NP. In addition, circ_0005075 significantly rescued NOTCH2 expression, which could be repressed by miR-151a-3p (Zhang Y. et al., 2021). Li et al. (2020) found that circZNF609 promoted the expression of inflammatory factors to exacerbate NP progression *via* the miR-22-3p/Enolase 1 (ENO1) axis in the CCI rat model. It was found that cZRANB1 mediated lysophosphatidic acid receptor 3 (LPAR3) expression *via* sponging miR-24-3p. MiR-24-3p regulated Wnt5a/β-Catenin signaling levels to promote NP progression *via* targeting LPAR3 in CCI rats (Wei et al., 2020).

Skin samples collected from patients with postherpetic neuralgia (PHN) were studied using miRNA and circRNA microarray to detect and analyze their expression profiles. Three hundred seventeen miRNAs differed in their expression between PHN-affected skin and normal skin. Only 13 of them showed fold change > 10 in the PHN skin. Bioinformatics analyses including gene ontology and Kyoto Encyclopedia of Genes and Genomes pathway were conducted to predict the mRNA targets of different miRNAs and also to study the function of these miRNAs. There are 85 pathways that had significant quantities of target miRNAs. This was the first study to research the different expression levels of miRNA and circRNA in the PHN skin. These abnormally expressed transcripts may be potential therapeutic targets to treat PHN (Cao et al., 2019).

CircRNAs associated with NP are listed in Table 3.

**TABLE 3:** CircNAs associated with neuropathic pain.

Models	circRNAs	Expression	Tissue	Target gene	References
DNP	circ HIPK3	↑	DRG	miR-124	Wang L. et al., 2018
SNI	circ AnKsla	↑	Spinal cord	VEGFB	Zhang S.B. et al., 2019
CCI	ciRS-7	↑	L4-L5 Spinal cord	miR-135a-5p	Cai et al., 2020
CCI	circ-0005075	↑	L4-L6 dorsal spinal cord	miR-151a-3p/NOTCH2	Zhang Y. et al., 2021
CCI	circ ZNF609	↑	L4-L6 dorsal spinal cord	miR-22-3p/ENO1	Li et al., 2020
CCI	circ ZRANB1	↓	L4-L6 dorsal spinal cord	Wnt5a/β-catenin, miR-24-3p/LPAR3	Wei et al., 2020

*NP, Neuropathic pain; circRNAs, circular RNAs; DRG, dorsal root ganglion; CCI, chronic constriction injury; DNP, diabetic neuropathic pain; L4, lumbar 4; L5, lumbar 5; L6, lumbar 6; VEGFB, vascular endothelial growth factor B; ENO1, enolase 1; LPAR3, lysophosphatidic acid receptor 3.*

## The Limitation in Neuropathic Pain Research

It has been widely reported that aberrant expression of ncRNAs exists after NP injury, and these differentially expressed ncRNAs are considered to be potential biomarkers for the diagnosis, assessment, treatment, prediction, and prognosis of NP. All these studies indicate that ncRNAs may act as potential biomarkers for clinical utility in patients with NP. Existing studies are limited to cell or animal models. Moreover, there are some differences between *in vitro* and *in vivo* experiments, and these differences are unpredictable. Therefore, these data are unpractical to be transformed and applied to the human body, which is still a challenge. Therefore, further studies need to focus on clinical trials to provide more validated evidences that ncRNAs can be used in the diagnosis, treatment, and prognosis of NP (Wu W. et al., 2019; Song et al., 2020).

## Conclusion

NP is a common disease in clinic, which seriously degenerates the quality of life in patients. The studies about the role of ncRNAs in NP are still at a preliminary stage. In the present study, some ncRNAs in the damaged peripheral nerve, DRG, and SDH may have a significant effect on the NP, but the detailed mechanisms of how the majority of these ncRNAs contribute to this disorder need more explorations. The aforementioned studies suggest that ncRNAs are endogenous promoters of chronic pain in the peripheral and central nervous system. ncRNAs may have potential to be used as biomarkers and new drug targets to treat NP. ncRNAs are supposed to have multiple and specific downstream targets due to their characters. However, the functions and mechanisms of these ncRNAs are needed to be elucidated in further studies.

## Author Contributions

WT designed the review and provided financial support. CH, MH, and QX collected the data from publications. CH developed the database, wrote the manuscript, and edited the final text. All authors read and approved the final manuscript.

## Conflict of Interest

The authors declare that the research was conducted in the absence of any commercial or financial relationships that could be construed as a potential conflict of interest.

## Publisher’s Note

All claims expressed in this article are solely those of the authors and do not necessarily represent those of their affiliated organizations, or those of the publisher, the editors and the reviewers. Any product that may be evaluated in this article, or claim that may be made by its manufacturer, is not guaranteed or endorsed by the publisher.

## Abbreviations

NP: Neuropathic pain
ncRNAs: non-coding RNAs
lncRNAs: long non-coding RNAs
DRG: dorsal root ganglion
UTR: untranslated region
pSNL: partial sciatic nerve ligation
SNI: spared nerve injury
CXCR4: chemokine CXC receptor 4
TXNIP: thioredoxin-interacting protein
NLRP3: NOD-like receptor protein 3
CCI: chronic constriction injury
HMGB1: high-mobility group box 1 protein
CXCL13: C–X–C motif chemokine 13
Cav1.2-LTC: L-type calcium channel
SIRT1: sirtuin1
Tregs: regulatory T cells
SDH: spinal dorsal horn
IRAK1: inhibiting interleukin-1 receptor-associated kinase 1
TRAF6: tumor necrosis factor receptor-associated factor 6
TIR: toll/interleukin-1 receptor
STAT3: signal transducer and activator of transcription 3
SOCS3: suppressor of cytokine signaling 3
NF-kB: nuclear factor-kappa B
p38 MAPK: p38 mitogen-activated protein kinase
SOCS1: suppressor of cytokine signaling 1
SN: sciatic nerve
BDNF: brain-derived neurotrophic factor
Cx43: connexin 43
BAMBI: bone morphogenetic protein and membrane-bound inhibitor
TGF- β: transforming growth factor- β
ZEB1: zinc finger E box binding protein-1
MeCP2: Methyl CpG binding protein 2
NOX4: NADPH oxidase 4
SCI: spinal cord injury
TLR8: toll-like receptor 8
ERK: extracellular regulated protein kinases
DNP: diabetic neuropathic pain
TRPV1: transient receptor potential vanilloid type 1
P2 × 7: P2X purinoceptor
SGCs: satellite glial cells
L5: lumbar 5
CDK6: cyclin-dependent kinase 6
CRNDE: colorectal neoplasia differentially expressed
XIST: X inactive specific transcript
TNFAIP1: tumor necrosis factor alpha-induced protein 1
COX-2: cyclooxygenase-2
TNF- α: tumor necrosis factor- α
IL-1 β, MALAT1: interleukin-1 β metastasis-associated lung adenocarcinoma transcript 1
NAT: natural antisense transcript
circRNAs: circular RNAs
PHN: postherpetic neuralgia
PDK4: pyruvate dehydrogenase kinase 4
RAP1A: Ras-associated protein 1A
SLC17A6: [solute carrier family 17 (sodium-dependent inorganic phosphate cotransporter), member 6]
NEAT1: nuclear paraspeckle assembly transcript 1
AQP4: aquaporin-4
MIP-1 α: macrophage inflammatory protein-1 alpha and CCR1, C–C chemokine receptor type 1
AMPK: AMP-activated protein kinase
EZH2: enhancer of zeste homolog 2
VEGFB: vascular endothelial growth factor B
ENO1: enolase 1
LPAR3: lysophosphatidic acid receptor 3
BACE1: β-site amyloid precursor protein-cleaving enzyme 1.
